# Construction of DNA Hemicatenanes from Two Small Circular DNA Molecules

**DOI:** 10.1371/journal.pone.0119368

**Published:** 2015-03-23

**Authors:** Claire Gaillard, François Strauss

**Affiliations:** Muséum National d'Histoire Naturelle, Paris, France; University of Helsinki, FINLAND

## Abstract

DNA hemicatenanes, one of the simplest possible junctions between two double stranded DNA molecules, have frequently been mentioned in the literature for their possible function in DNA replication, recombination, repair, and organization in chromosomes. They have been little studied experimentally, however, due to the lack of an appropriate method for their preparation. Here we have designed a method to build hemicatenanes from two small circular DNA molecules. The method involves, first, the assembly of two linear single strands and their circularization to form a catenane of two single stranded circles, and, second, the addition and base-pairing of the two single stranded circles complementary to the first ones, followed by their annealing using DNA topoisomerase I. The product was purified by gel electrophoresis and characterized. The arrangement of strands was as expected for a hemicatenane and clearly distinct from a full catenane. In addition, each circle was unwound by an average of half a double helical turn, also in excellent agreement with the structure of a hemicatenane. It was also observed that hemicatenanes are quickly destabilized by a single cut on either of the two strands passing inside the junction, strongly suggesting that DNA strands are able to slide easily inside the hemicatenane. This method should make it possible to study the biochemical properties of hemicatenanes and to test some of the hypotheses that have been proposed about their function, including a possible role for this structure in the organization of complex genomes in loops and chromosomal domains.

## Introduction

**Fig 1:**
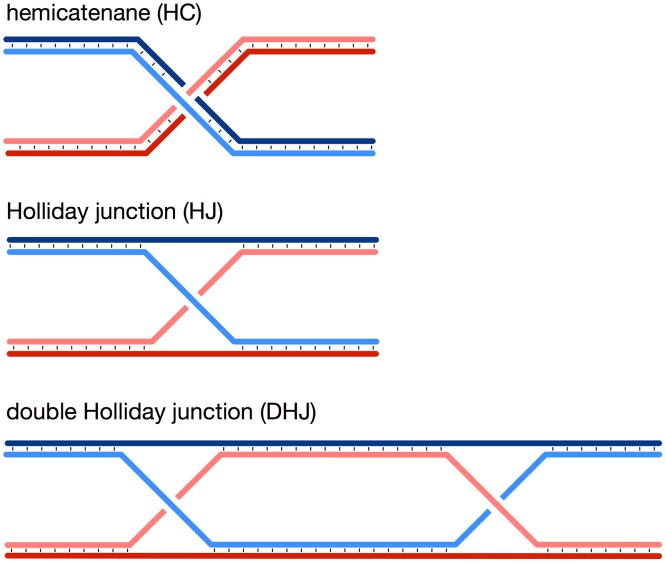
Schematic representation of simple DNA junctions.

DNA hemicatenanes consist of a junction of two double-strand DNA molecules, in which one strand of one duplex passes between the two strands of the other duplex, and reciprocally, as schematically represented in Fig. 1. Unlike in most other four-stranded DNA structures, such as the Holliday Junction (HJ, Fig. 1) and the Double Holliday Junction (DHJ, Fig. 1), a characteristic of the hemicatenane is that the joined DNA duplexes are fully distinct, no sequence homology being required between the two DNA molecules to form a hemicatenane.

The Holliday junction and double Holliday junction have been deeply studied, given their importance in many DNA transactions such as homologous recombination or repair of double-strand DNA breaks, or as building blocks in DNA nanotechnology [1]. Technically, most of these studies were based on the possibility to prepare this junction despite its marked instability. This was performed either by association of DNA molecules able to migrate in one direction only [2]; or by building a DHJ between two circular DNA molecules thus making its spontaneous dissociation topologically impossible [3,4]; or, in most studies, by using synthetic oligonucleotides of appropriate sequences to prepare cruciforms, i.e. stable four-way junction analogs that were unable to migrate (for a review see e.g. [5]).

Hemicatenanes have been much less studied experimentally, yet many publications have suggested that they might play important roles in various DNA transactions. Replication intermediates that were suggested as possible hemicatenanes were observed e.g. during replication of the genome of SV40 virus ([6], and [7] as reanalyzed in [8]), in plasmid replication in Xenopus egg extracts [9], or during replication in yeast [10,11]. In double-strand-break repair, a hemicatenane has been proposed to be the final intermediate in the convergent migration of double Holliday junctions, in a process catalyzed by a type IA topoisomerase, TopIIIα in human cells (Top3 in yeast), in association with the RecQ helicase BLM (Sgs1 in yeast) and the RMI1-RMI2 complex (Rmi1 in yeast) [12–15]. It has also been suggested [16–18] that an appropriate processing of hemicatenanes is essential for genome stability. In addition, our previous work showed that four-stranded DNA structures formed in vitro by DNA fragments containing tracts of the poly(CA)·poly(TG) sequence (CA-microsatellites) consisted in DNA loops with a hemicatenane at their base [19] and were tightly bound by nuclear protein HMGB1 in vitro [20,21]. This led us to suggest that complex genomes might be organized in chromosomal loops maintained by topological DNA knots, possibly hemicatenanes, a hypothesis with original and interesting implications on the control of genome function during development and differentiation [22,23].

At this point it became clear, first, that the most direct way to test some of the hypotheses raised would require the ability to construct and purify hemicatenanes in order to study their biochemical properties; and, second, that hemicatenanes should no longer be prepared by looping of a single repetitive DNA molecule but instead by association of two distinct DNA molecules of any nucleotide sequences. As the stability of hemicatenanes of two linear molecules seemed very unlikely, both on a theoretical basis and according to preliminary experiments made by cutting hemicatenated loops inside the loops (our unpublished data), we set out to build hemicatenanes of two circular DNA molecules.

A first step in that direction had been previously made by Bucka and Stasiak [24], who prepared catenanes of two single stranded circles of 60 nucleotides possessing a sequence complementarity along 12 bases. We wished to prepare hemicatenanes of two DNA sequences with no significant sequence homology, however. To avoid the presence of kinks in the double strand DNA double helix, we chose to use DNA circles with sizes well above the 150 bp persistence length of double stranded DNA [25].

Here we describe the construction of hemicatenanes from two DNA minicircles of 235 and 216 bp, and the first characterization of the products obtained.

## Materials and Methods

### Water

Due to unexplained random degradation of single stranded DNA observed in the early stages of this work, quartz-bidistilled water was used in all further experiments.

### Enzymes

All enzymes were from New England Biolabs, with the exception of wheat germ topoisomerase I (Promega in early experiments, later Inspiralis), calf thymus topoisomerase I (Invitrogen), and nicking endonuclease Nb.Mva1269 I (Fermentas), which was preferred to its isoschizomer Nb.BsmI for its optimal digestion temperature of 37°C, as opposed to 65°C for Nb.BsmI.

Note: care was taken not to overdigest with Nb.BtsI, as this nicking enzyme tended to show non-specific star activity when used at high concentration (data not shown).

### Plasmids

**Fig 2:**
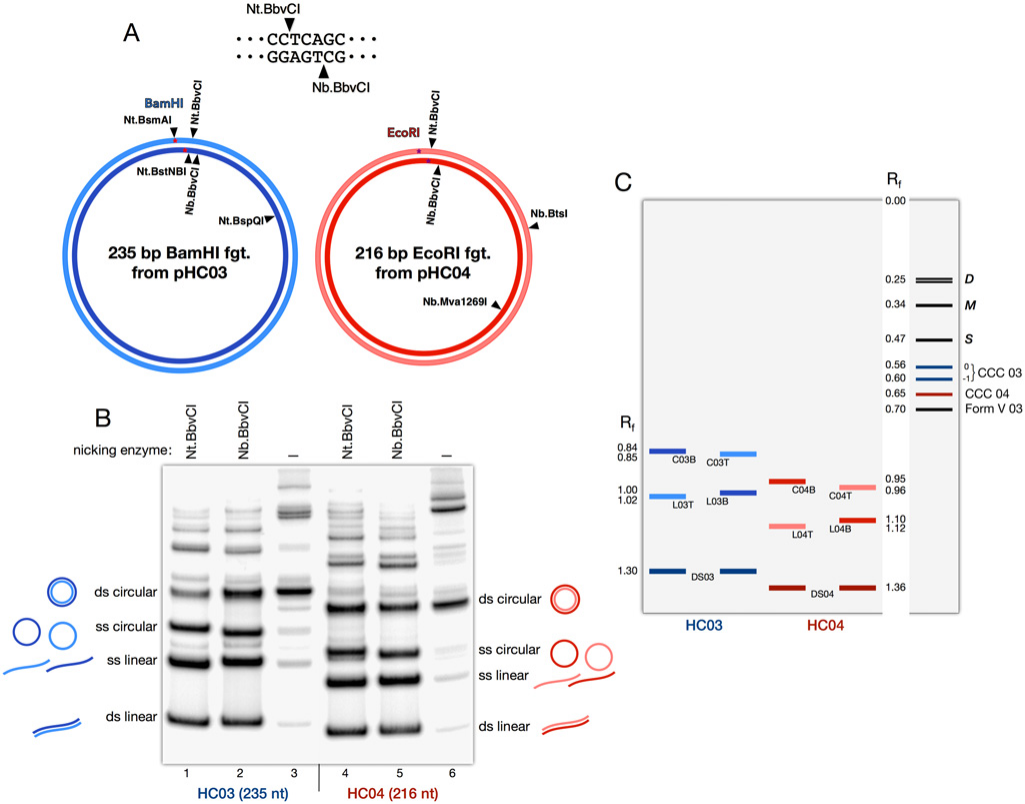
DNA minicircles used to build hemicatenanes. **A**. Maps of the two small DNA circles used in the present work. A 235 bp BamHI fragment and a 216 bp EcoRI fragment, respectively extracted from plasmids pHC03 and pHC04, were ^32^P end-labeled and circularized. Each DNA circle contained sites for several nicking endonucleases, as indicated. Nt.BbvCI and Nb.BbvCI sites, shown at the top of the panel, were present on both circles and were used for purification of linear or circular single strands (see panel B). The other nicking endonucleases were used for probing the arrangement of DNA strands in the final product. **B**. Preparative gel of the linear and circular strands of both minicircles. DNA circles were digested with nicking endonucleases Nt.BbvCI (lanes 1, 4) or Nb.BbvCI (lanes 2, 5), denatured with alkali, and loaded on a preparative polyacrylamide gel. In the two other lanes (lanes 3, 6), DNA circles were not nicked but were also treated with alkali and loaded on the gel, in order to purify double-stranded covalently closed DNA circles. The numerous minor bands on the gel correspond to digestion products of linear and circular dimers and longer oligomers of the DNA fragments, which are unavoidable side products of the ligation reactions. Abbreviations ds and ss refer to double-stranded and single-stranded forms. **C**. Drawing of a 4% polyacrylamide preparative gel, showing the main bands encountered in the present work, with their relative mobilities R_f_ relative to the linear bottom strand of circle 03, arbitrarily taken as reference. Nomenclature: for the single strands, L refers to a linear strand, C to a circular strand; 03 or 04 is the number of the plasmid of origin (pHC03 or pHC04), T and B refer to the Top or Bottom strand, as determined by the presence of a site for Nt.BbvCI (Top) or Nb.BbvCI (Bottom) nicking enzyme. For example, L03B refers to the linear strand of circle 03 that contains a site for Nb.BbvCI nicking enzyme. Shown on the right: *D*: double strand hemicatenanes, *M*: hemicatenane of one single strand with one double strand, *S*: catenane of two single stranded circles (see Text); CCC: covalently closed circles (topoisomers 0 and -1 of CCC 03 are indicated); Form V: association of two complementary single-stranded circles into a paranemic double-stranded circle (linking number L_k_ = 0).

DNA fragments used in the present work originated from two plasmids, pHC03 and pHC04, which were constructed from the classical cloning vector pTZ19R as follows: the EcoRI-HindIII fragment of pTZ19R, which contains the multiple cloning sites, was replaced by artificial fragments made by ligation of synthetic oligonucleotides of appropriate sequences, and designed to contain several restriction sites plus recognition sites for site-specific nicking endonucleases, as shown on Fig. 2A. The synthetic replacement sequences used have been deposited at the European Nucleotide Archive under accession numbers HG423213 and HG423214 for pHC03 and pHC04, respectively. Both plasmids were amplified in E. coli strain DH5α, purified using a plasmid purification kit (Qiagen), and verified by sequencing.

### DNA purification and ethanol precipitation

After each enzymatic step, DNA was purified and concentrated by the following protocol (with the exception of dephosphorylation with calf intestine phosphatase, an enzyme which must be inactivated with phenol). This protocol was also used to remove intercalated ethidium bromide after electroelution, when present.

Raise the volume of the DNA sample to 300 μL with 10 mM Tris-HCl, 1 mM EDTA, pH 7.6 (TE buffer), add 20 μL of 20% SDS and 80 μL of 5M NaCl, mix and place at 37°C for a few moments to dissolve the SDS precipitate, add 1 vol. of chloroform-isoamyl alcohol (24:1 v/v), vortex at top speed for 2 min, place the tubes on ice for 5 min to precipitate the SDS, centrifuge at 4°C for 5 min at ~15,000 x g, recover the aqueous upper phase, add 10 μL (25 μg) linear polyacrylamide carrier [26], add 1 mL ethanol, mix well by inverting the tube several times, leave on ice for 10 min, centrifuge 10 min at 15,000 x g, discard the liquid, spin the tubes for 20 sec at low speed and carefully remove the rest of liquid with the tip of a micropipette, dry the pellet by leaving the tubes open for 5 min in a 37°C heating block, redissolve the pellet.

### DNA storage

Single stranded DNA dissolved in TE buffer was found to be readily lost by adsorption to the walls of polypropylene tubes, even "low-binding" tubes. To overcome this, the non-ionic detergent Triton X-100 was systematically added to single-stranded DNA at a concentration of 0.05%, which very efficiently suppressed tube walls adsorption. Routinely, single-stranded DNA was dissolved in 10 mM Tris-HCl, 1 mM EDTA, 50 mM NaCl, 0.05% Triton X-100, pH 7.6 (TENT buffer), and stored at 4°C.

### Single stranded DNA and Triton X-100

**Fig 3:**
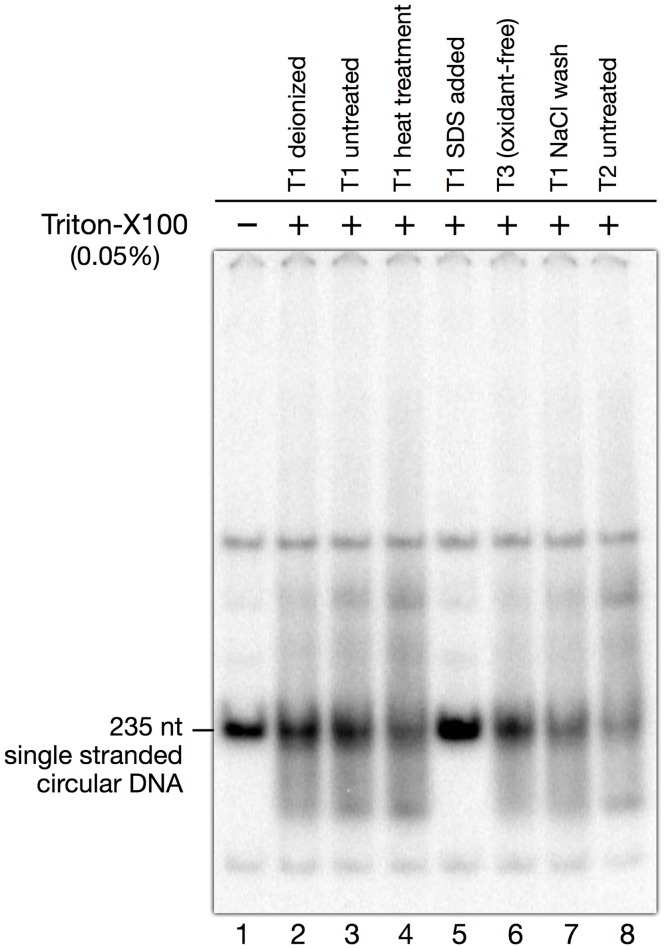
Effect of Triton X-100 on the migration of single stranded DNA in polyacrylamide gels. The gel illustrates the smearing of single stranded DNA when loaded on polyacrylamide gels in the presence of Triton X-100. The DNA sample was the same in all lanes and contained a labeled single stranded circle of 235 nucleotides. Two minor bands that show no smearing most probably correspond to contaminating double stranded DNA, as smearing was always observed with many different single strands. Three different brands of Triton-X100 denoted T1–T3 were tested at a final concentration of 0.05%: T1 and T2 were ordinary grade, T3 was highly purified, oxidant-free Triton-X100, sold in glass ampules sealed under nitrogen (Pierce). Lane 1: control, no Triton-X100 added; lane 2: deionized T1; lane 3: T1, untreated; lane 4: T1, heat-treated; lane 5: T1, SDS added at a concentration of 0.005%; lane 6: T3, untreated; lane 7: T1, washed with a 5M NaCl solution; lane 8: T2, untreated. Note that addition of SDS to the samples before loading the gel was a very efficient way to suppress smearing (lane 5).

While preventing adsorption on tube walls, Triton-X100 was found to present an unexpected problem specific to single stranded DNA. Single stranded DNA loaded at neutral pH on 4% polyacrylamide gels showed smears instead of bands (Fig. 3). Deionization of Triton-X100 with mixed-bed ion exchange resin, or use of highly-purified Triton-X100 sold in glass ampules sealed under nitrogen (Pierce), did not significantly change the result (Fig. 3). We did not identify the cause of this problem, and we do not know whether it is due to Triton-X100 itself or to a contaminant. Neither did we test other non-ionic detergents, which might not show the same effect. In practice, the problem was overcome by adding SDS to 0.005% to all samples loaded at neutral pH on polyacrylamide gels. This low concentration of SDS was found to very efficiently suppress any smearing (Fig. 3, lane 5).

### Polyacrylamide gel electrophoresis

Unless indicated otherwise, DNA was loaded on 4% polyacrylamide gels (acrylamide:bis-acrylamide ratio 29:1 w/w) in 0.5 x TBE buffer (Gibco; 10 x TBE buffer is 1.0 M Tris, 0.9 M Boric acid, 0.01 M EDTA) run at room temperature at a constant voltage of 5 V/cm. These conditions were found by trial and error to give a good separation of the different forms, single strand and double strand, circular and linear, for the two fragments of 216 and 235 bp used. Electrophoresis was for 4 hr to separate simple DNA species, or 5.5 hr for hemicatenanes.

### Preparation of samples for electrophoresis

Depending on the experiments, DNA was loaded on gels either in native conditions at neutral pH, or denatured by alkali.

Electrophoresis of native samples: samples loaded at neutral pH contained 2% glycerol, plus 0.005% SDS as indicated in the previous paragraph; no tracking dye was used.

Electrophoresis of denatured samples: alkali denaturation was more reliable and more convenient than denaturation by temperature (heating the samples was found to induce important losses of material, possibly because Triton X-100 separates from water to form a distinct phase above its cloud point of 64°C). To DNA in TENT buffer, we added ¼ vol. of a 10% glycerol, 0.2 N NaOH solution, which raised the pH to 12.6, a value sufficient to denature DNA, but not too high so that circular DNA molecules were not irreversibly denatured [27]. In that way, covalently-closed double-stranded DNA circles migrated in their native state and were separated from nicked circles, which were denatured. Adding SDS was not necessary, as smearing due to Triton-X100 did not appear in samples loaded at alkaline pH.

### DNA fragments

Plasmid pHC03 was digested by BamHI, pHC04 by EcoRI, so as to obtain DNA fragments of 235 and 216 bp, respectively (Fig. 2), which were used to prepare the two circular DNA molecules to be hemicatenated. Both plasmids were found to require high concentrations of restriction enzymes for complete digestion. Conditions for completeness were as follows: 250 ng/μL plasmid, 1 unit/μL enzyme (New England Biolabs high-concentration high-fidelity enzymes), incubation for 3 hr at 37°C. Fragments were end-dephosphorylated by addition of 0.1 units/μL calf intestine phosphatase at the beginning of the third hour of digestion.

After control of digestion on an agarose gel, the phosphatase was inactivated by extraction with phenol, phenol-chloroform (1:1 v/v), and chloroform-isoamyl alcohol (24:1 v/v). DNA was precipitated with ethanol, redissolved in 300 μL of 10 mM Tris, 1 mM EDTA, pH 7.6, glycerol added to 2.5%, and samples loaded on a 4% preparative polyacrylamide gel (acrylamide:bisacrylamide 29:1) in 40 mM Tris, 20 mM Na Acetate, 1 mM EDTA, pH 7.8. After 4 hr of migration at 5 V/cm, the gel was stained with ethidium bromide, gel slices containing the 235 and 216 bp fragments were cut from the gel under 365 nm UV illumination, DNA was electroeluted, chloroform-extracted, precipitated as described above, and dissolved in TE buffer. The concentration of DNA fragments was measured with a NanoDrop spectrophotometer.

### DNA labeling

DNA fragments were end-labeled with ^32^P using T4 polynucleotide kinase and [ɣ-^32^P]ATP. As our objective was to build a four-stranded structure, it was found important not to use ATP of high specific activity. The ideal would be to have a single ^32^P atom in each structure built, as the decay of a ^32^P atom into ^32^S quickly result in a nick due to the instability of the sulfodiester bond [28]. If a structure contains several ^32^P atoms, the decay of any one of them leads to the formation of a labeled contaminant, whereas when the four-stranded structure contains a single ^32^P atom the decay product will not be radioactive and will not be visible upon autoradiography (note that one should remain aware of the fact that it is present, a phenomenon that is unavoidable when using radioactive isotopes).

For this reason, [ɣ-^32^P]ATP (Perkin-Elmer, 3000 Ci/mmol, i.e. containing ~ ⅓ ^32^P and ~ ⅔ ^31^P at position ɣ) was diluted with non-radioactive ATP in a proportion such that the mixture contained 10% radioactive ATP only. Thus, the probability that a four-stranded structure would contain two ^32^P atoms was low, and decay contaminants were hardly visible.

After labeling, the DNA fragments were extracted with chloroform-isoamyl alcohol as described above, precipitated twice with ethanol to get rid of the unincorporated radioactivity, and redissolved in TE buffer.

### Circularization of DNA fragments

Labeled DNA fragments were circularized with T4 DNA ligase in a volume of 400 μL, at 16°C, with 1000 units (2.5 μL) of T4 DNA ligase. To favor the circularization of DNA fragments into monomeric circles, we found that the concentration of DNA fragments had to be lower than 0.25 ng/μL. And to obtain large amounts of DNA circles, 1 μg or more, while keeping the volume of ligation small and manageable, we adapted the ingenious protocol of Prunell et al. [29]. 2 μL DNA aliquots, containing no more than 100 ng of fragment each, were added to the ligation mix every 10 min. In that way the concentration of linear fragments remains always low, since the circular ligation products that form continuously do not participate in the ligation reaction. When all the DNA fragments had been added, ligation was continued for 1 hr, then SDS added to 1%, NaCl to 1M, DNA extracted with chloroform-isoamyl alcohol and precipitated with 1 vol. absolute ethanol (using 1 vol. instead of the more usual 2.5 vol. avoids precipitation of the ATP in the ligation buffer, with no change in precipitation efficiency when linear polyacrylamide carrier is present).

### Purification of single-stranded DNA

Both DNA fragments used to prepare the DNA circles contained a site for the restriction endonuclease BbvCI, allowing us to specifically cut either strand of either circle with nicking endonucleases Nt.BbvCI or Nb.BbvCI (Fig. 2A). After digestion with the appropriate nicking enzyme, DNA was denatured with NaOH as described above and loaded onto a 4% preparative polyacrylamide gel in 0.5xTBE. As an example, Fig. 2B shows the phosphorimager image of such a gel, with the circularization of both fragments from pHC03 and pHC04 (lanes 3 and 6, respectively), and the result of nicking both preparations of DNA circles with either nicking enzyme, as indicated at the top. Fig. 2C shows a drawing representing a gel with the major DNA species encountered in the present work, and indicating the relative mobilities Rf of all the bands relative to the linear strand L03B arbitrarily taken as reference. Bands containing the circular or linear strands of the DNA circles were cut, DNA electroeluted, ethanol precipitated, redissolved in TENT buffer, and stored at 4°C.

### Circularization of single stranded DNA molecules

Synthetic oligonucleotides of 37 bases were used as ligation-helpers, of which 25 bases were complementary to the 3' end and the remaining 12 bases to the 5' end of the DNA strand to be circularized. The 12 bases formed a 5'-protruding sticky end that was sufficient to promote efficient circularization by ligation, with little formation of linear oligomers.

### Hybridization of DNA strands

DNA strands in TENT buffer were mixed in a 1.5 mL microtube, NaCl added to 250 mM, and samples incubated for 6 to 12 hr at 52°C. Tubes were placed on a foam rack and floated in a water bath under an inverted beaker used as a wet diving-bell, which allows one to incubate samples of extremely small volumes for extended periods of time with no trace of condensation on tube caps.

### Renaturation of covalently-closed DNA circles with topoisomerase I

**Fig 4:**
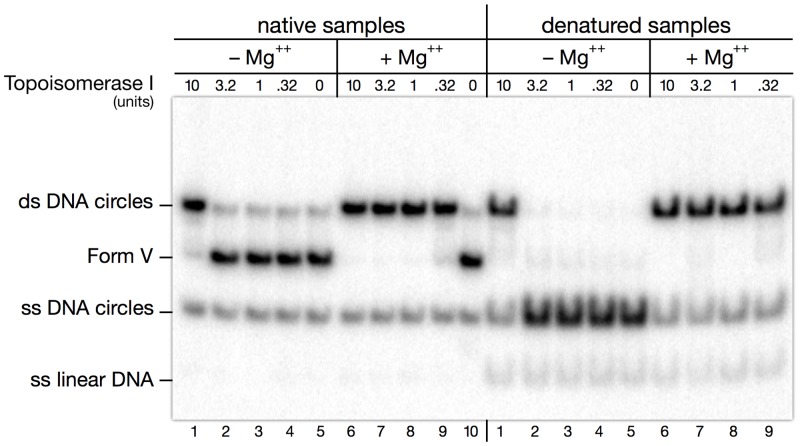
Annealing of complementary single stranded circles ("Form V" DNA) by wheat germ topoisomerase I. The two complementary single stranded DNA circles prepared from minicircle HC03 were mixed and allowed to hybridize into Form V DNA, then annealed into a regular B-form covalently-closed DNA circle by incubation with various amounts of wheat germ topoisomerase I, either in the absence or in the presence of Mg^++^ in the incubation buffer. Samples were then split in two parts and analyzed on a polyacrylamide gel, either native (left), or denatured by alkali (right). Note that a strong stimulation of annealing by Topoisomerase I is observed in the presence of magnesium (compare lanes 1–4 with lanes 6–9).

Hybridization of two complementary single-stranded circles results in the formation of a double-stranded circular DNA molecule in which both strands are base-paired without being topologically linked (L_k_ = 0, Form-V DNA, [30]). Type IA or IB topoisomerases can catalyze the annealing of the paired strands into a B-form double helix and convert this structure into relaxed double strand circles [31,32]. To perform this reaction we used wheat germ topoisomerase, a commercially available type IB enzyme which as such does not dissociate single strand catenanes, and which was found to be slightly more efficient than calf thymus topoisomerase I for perfectly annealing two complementary single-stranded circles (data not shown). We observed an unexpected point however: whereas the relaxation activity of this enzyme does not require the addition of magnesium to the incubation buffer, annealing of single stranded circles was much more efficient when magnesium was present (Fig. 4). Annealing was thus performed in 50 mM Tris-HCl pH 7.6, 50 mM NaCl, 5 mM MgCl_2_, 1 mM DTT, 0.05% Triton X-100, 0.2 mM EDTA, for 2 hr at 37°C. Under these conditions, the DNA circles obtained by annealing the two circular strands were absolutely indistinguishable from those obtained by relaxation of the original circles by the same enzyme under identical conditions, as judged by the distribution of topoisomers on gels containing chloroquine (not shown).

### Construction of fully catenated DNA circles

Catenated single strand circles prepared as described in Results were hybridized with both complementary linear strands, and incubated with T4 DNA ligase to seal the nicks. The ligation products were fractionated on a preparative 4% polyacrylamide gel, the fully catenated circles were found to migrate at the same position as the upper band of hemicatenanes.

## Results

**Fig 5:**
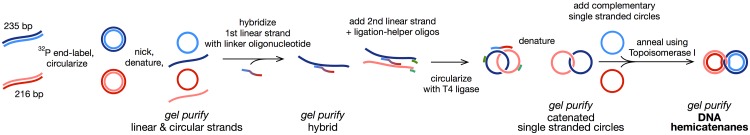
Strategy for the construction of hemicatenanes of two small DNA circles.

The strategy selected to construct hemicatenanes of two double-stranded DNA circles is schematically represented in Fig. 5. Two DNA fragments, sharing no significant sequence similarity, are purified, ^32^P end-labeled, and circularized with DNA ligase. Both double stranded circles are then nicked, each at a single specific site, denatured, and run on a preparative polyacrylamide gel in order to purify, for each circle, the single stranded circle and its linearized complementary strand.

The next step, inspired by previous work of Bucka and Stasiak [24], consists in the circularization of both linear strands while keeping them in close proximity with a synthetic oligonucleotide able to bind both sequences (Bucka and Stasiak used partial sequence homology between the two strands). This leads, in part, to the formation of a catenane between the two single-stranded circles, which is gel purified.

In the last step, the circular strands purified earlier are hybridized to the single strand catenane, then annealed using wheat germ topoisomerase I, resulting in the formation of a hemicatenane between the two initial DNA circles, which is gel purified.

**Fig 6:**
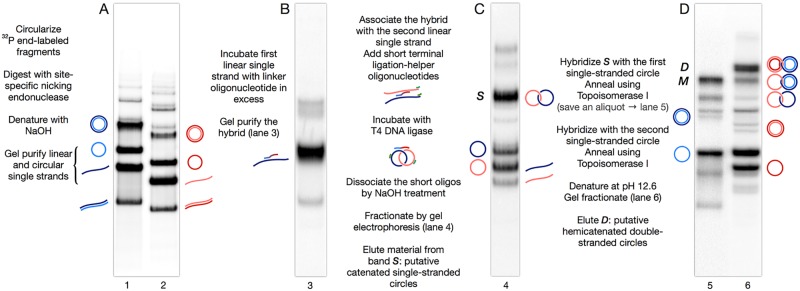
Construction of hemicatenanes. Shown are the four preparative polyacrylamide gels involved in the purification of hemicatenanes following the protocol of Fig. 5. A: Purification of one linear strand and one circular strand of each DNA circle. B: Purification of the hybrid between the linear strand of the first circle and the synthetic linker oligonucleotide. C: Result of the circularization of the linear single strands, with the new band, *S*, being expected to contain catenated single-stranded circles. D: Result of annealing material from band *S* with one (lane 5) or both (lane 6) circular single strands that were prepared in A. A new band, *M* (lane 5), appears upon annealing with the first single stranded circle, plus a second band, *D* (lane 6), after annealing with the second single stranded circle. Band *D* is expected to contain hemicatenated circles. Panel A shows the preparative gel for the separation and the purification of the strands of both circles, one strand linear and one strand circular for each circle. Panel B shows the purification of the hybrid between the linear strand of the first circle and the synthetic oligonucleotide linker that will serve as a bridge between the two linear strands and allow them to catenate, at least partially, when circularized at the next step (no catenanes form if strands are not held in close proximity by the linker oligo, data not shown). Gel purification of the hybrid between the first strand and the oligonucleotide is necessary to get rid of the excess oligonucleotide which would otherwise interfere with the association of the second strand. The purified hybrid is then associated with the second linear strand, which hybridizes to the linker oligo. Short ligation-helper synthetic oligonucleotides that bind to the 3' ends of both strands are also added. After incubation with DNA ligase, a preparative gel electrophoresis (Panel C) shows that little of the linear strands remains, while a significant amount of the strands is now found as single circles, and most noteworthy a new band appears, labeled *S*, which is expected to consist in catenanes of the two single stranded circles, as it contains material that migrates much more slowly than single circles.

Fig. 6 shows the application of this strategy in practice, taking into account all experimental technical details described in Materials and Methods. The purification, labeling, and circularization of DNA fragments are straightforward and not shown here.

This material is eluted from the gel and associated with the circular strand of the first circle (which was purified in A), the hybrid is then incubated with topoisomerase I to anneal the two strands of the first circle. The circular strand of the second circle is then added, and in turn annealed using topoisomerase I as above. The product is denatured and fractionated on a preparative gel. This shows the stepwise appearance of a first slow-migrating band, M (lane 5) after annealing of the first circular strand, then a second band, D (lane 6) after annealing of the second strand. Material present in band D, presumed to consist in hemicatenanes of the two double-stranded circles, is electroeluted for further analysis and characterization. Note that band D runs as a double band, which will be explained in Discussion.

**Fig 7:**
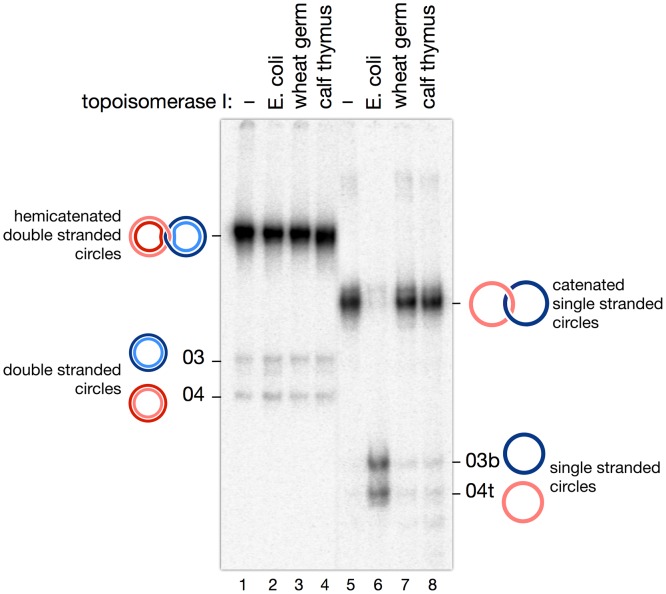
Assay of topoisomerases I with double strand hemicatenanes and single strand catenanes. Material from bands *D* and *S* (Fig. 6C & D), expected to correspond respectively to hemicatenanes and single strand catenanes, were incubated with topoisomerase I from E. coli, wheat germ, and calf thymus. E. coli topoisomerase I, a type IA topoisomerase, is able to decatenate the single strand circles as expected, but has no effect on double strand hemicatenanes. The other two enzymes, type IB topoisomerases, show no activity on either substrate.

Experiments were then performed in order to confirm the actual nature of material eluted from bands D and S (putative hemicatenanes and single strand catenanes, respectively). Fig. 7 shows an analysis using type I topoisomerases: E. coli topoisomerase I (a type IA enzyme), wheat germ and calf thymus topoisomerases I (type IB enzymes). Interestingly, none of these topoisomerases have any effect on D. With S, E. coli topoisomerase decatenates the two single strand circles, whereas both type IB enzymes have no effect. This is exactly what was expected for a single strand catenane, E. coli topoisomerase I being able to decatenate single strands [33], whereas type IB enzymes act as swivelases and cannot catalyze this reaction (the exact mode of catenation of the two circles may be more complex than schematically represented on the Figure, however; see Discussion).

**Fig 8:**
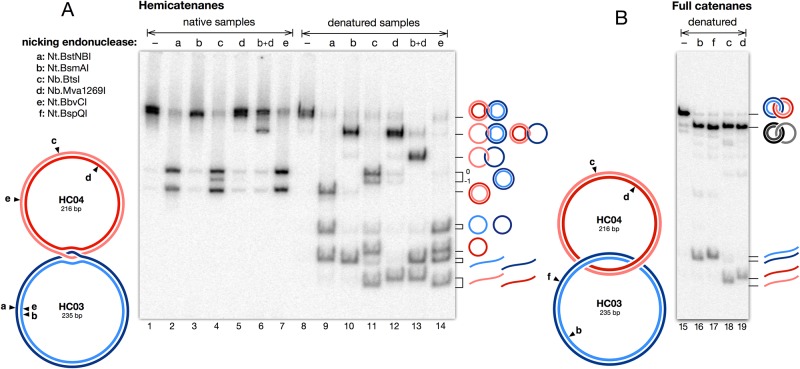
Probing the arrangement of strands with site-specific nicking endonucleases. A: The drawing represents the expected structure of hemicatenanes prepared according to the protocol used in the present work, showing the cutting sites for nicking enzymes and the relative arrangement of the four strands at the hemicatenated junction. The gel shows the result of nicking the material eluted from band *D* (Fig. 6D, lane 6) with the indicated nicking enzymes. After digestion, samples were split and analyzed either as native material (left half of the gel) or denatured by alkali (right half). The locations of all DNA species, as determined in previous experiments, are indicated schematically on the right side of the gel. B: A similar experiment was performed with fully catenated circles prepared as described in Materials and Methods. As in A, nicked samples were analyzed either native (not shown, as nicking produced no visible effect on the gel) or denatured.

A detailed analysis of the material from band D was then performed using nicking endonucleases and is shown in Fig. 8A. The material was digested with site-specific nicking endonucleases having cutting sites on either strand of either circle, as represented on the drawing. To interpret the results, one should remember that, in a hemicatenane, the DNA strands are not equivalent: for each duplex, one strand (internal strand) is trapped between the two strands of the other duplex, while the other strand (external strand) lies on the outside of the junction.

The results obtained correspond very precisely to what was expected for two hemicatenated circles, whether samples are analyzed as native material (left half of the gel) or denatured by alkali (right part). A nick on an external strand (b,d) shows no visible effect in native samples, and forms band M in denatured samples. A nick on an internal strand (a,c) leads to dissociation into two double stranded circles (native samples), one of which is nicked and dissociates into a single stranded circle plus a linear strand when denatured. It is very interesting to note that a nick on an internal strand (a,c) leads to dissociation of the structure without any treatment other than incubation at 37°C during digestion with the nicking enzyme. This instability of the hemicatenane, which also points to a great mobility of the junction, will be discussed in detail in Discussion. Simultaneous nicking of both external strands (b+d) results, in denatured samples, in a band with a mobility that is precisely that of band S, the single strand catenanes. In native samples, a new band appears which has not yet been characterized but might correspond to a structure in which both circles have moved so that nicks have become positioned at the junction. Finally, simultaneous nicking of the external strand of one circle and the internal strand of the other (e) results in two separated nicked circles, again as expected.

For comparison, a control was made (Fig. 8B) by constructing the full catenane of the same two double stranded circles (see Materials and Methods), which migrates at the same position as hemicatenanes. On this material, a nick on any of the four strands produces no visible migration change on native samples (not shown), and when denatured leads to the formation of a band (Fig. 8B) that migrates very close to band M observed above, albeit slightly more slowly, and corresponds to the full catenane of a single stranded circle with a double stranded circle.

### Measurement of the unwinding of the double helix in hemicatenanes

**Fig 9:**
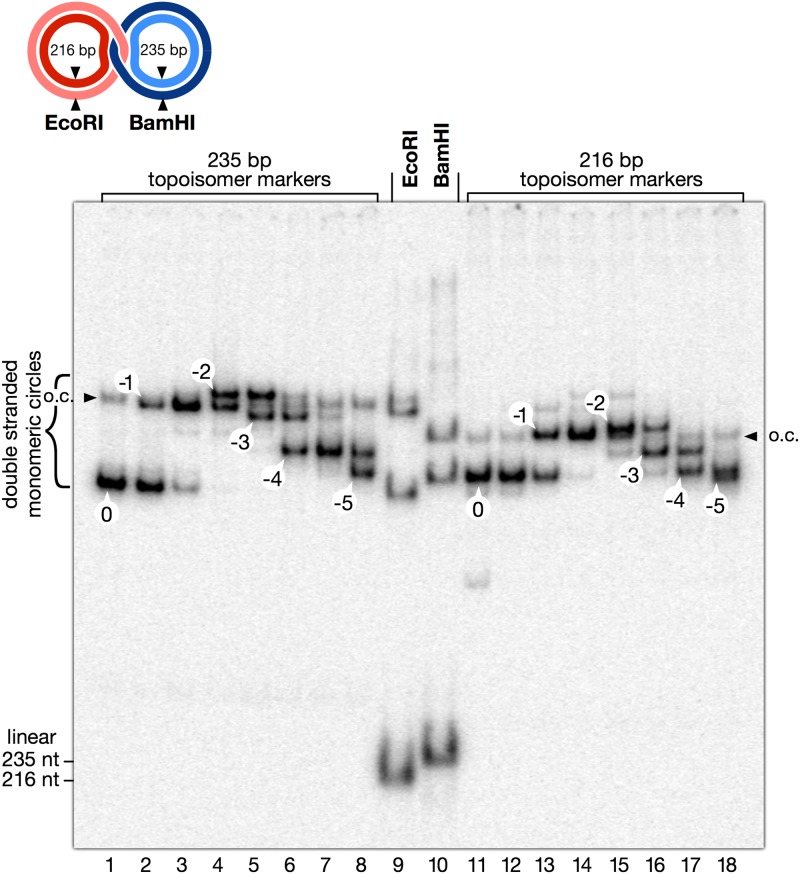
Unwinding of the DNA double helix in hemicatenanes.

In a hemicatenane, one should expect each of the DNA duplexes to be somewhat unwound by the single strand of the other duplex. This unwinding could be measured. One of the two circles was digested with a restriction enzyme and eliminated by alkali treatment. (Spontaneous dissociation of hemicatenanes upon linearization of one of the circles was very fast but never 100% complete, possibly due to to preferential pausing of the junction at specific sites or to a small amount of complex knots. Therefore treatment with alkali was the most efficient way to achieve complete dissociation). The remaining circle was then loaded on a polyacrylamide gel containing chloroquine (Fig. 9), and compared to a series of marker topoisomers prepared independently by incubation of the DNA circles with topoisomerase I in the presence of increasing amounts of ethidium bromide [34], under conditions strictly identical to the conditions used for construction of hemicatenanes. It is observed (Fig. 9, lanes 9,10) that circles released from hemicatenanes are underwound relative to individual circles prepared in the absence of ethidium bromide (lanes 1 and 11). For the larger circle, the distribution is about 45% of topoisomer 0, 45% of topoisomer-1, and 10% of-2. Similarly for the smaller circle, about 50% of topoisomer 0 and 50% of-1. The unwinding of the circles is therefore close to half a turn of the double helix, a value that seems quite compatible with a hemicatenane and will be discussed below.

DNA hemicatenanes, prepared as described and schematically represented at the top, were digested with EcoRI or BamHI and denatured by alkali to get rid of the cut circle. The remaining double strand circle was analyzed on a polyacrylamide gel containing chloroquine, alongside topoisomer markers which had been prepared independently by incubating isolated circles with topoisomerase I in the presence of various amounts of ethidium bromide. The markers on the gel are labeled according to the number of negative supercoils they contain relative to the main topoisomer obtained in the absence of ethidium, labeled 0 (markers contained a small amount of nicked circles, o.c., indicated by arrowheads). It is observed that circles released from hemicatenanes are underwound by an average of approximately half a double helical turn, relative to marker circles relaxed by topoisomerase under identical conditions.

## Discussion

**Fig 10:**
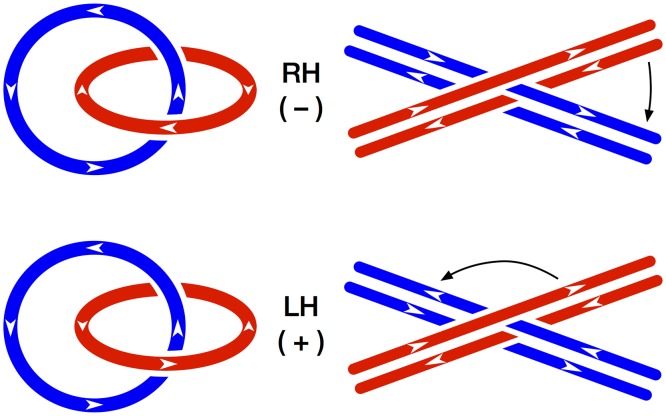
Chiral forms of single strand catenanes and hemicatenanes. Catenanes of single stranded DNA circles are represented on the left, hemicatenanes of double stranded DNA fragments on the right. Due to the 5'-3' polarity of DNA strands, these structures can exist as stereoisomers with clockwise (–) or counterclockwise (+) crossings [35], also called right-handed (RH) or left-handed (LH), respectively. Right-handed hemicatenanes can readily be identified (on drawings) by the fact that each pair of adjacent single strands cross as in the righ-handed DNA double helix, so that aligning the two duplexes would require rotating the top strand clockwise, to the right (arrow).

Our objective in the present work was to build DNA hemicatenanes, i.e. the simplest structure in which two double strand DNA molecules are associated by the passage of one strand of one duplex between the two strands of the other duplex, in the simplest manner, without any winding of the strands around each other at the junction. This structure is perfectly defined topologically, as long as the possibility is taken into account that it can exist under two chiral forms, right-handed or left-handed (Fig. 10).

Our results show that, using commercially available enzymes, this structure can be built in a carefully controlled bottom-up approach. The procedure yields a product with characteristics expected for hemicatenanes: it is made of two distinct DNA circles that are covalently linked and resistant to denaturation by alkali; the arrangement of strands, as probed with site-specific nicking endonucleases, is exactly as expected; each of the two circles is underwound by half a double helical turn, on average, relative to the same circle taken in isolation. Therefore the results obtained during the construction steps, combined with the characterization of the final product, indicate that we are indeed dealing with DNA hemicatenanes of two circular DNA molecules.

The method requires a series of steps including six enzymatic reactions and four purifications of various DNA species by preparative gel electrophoresis, with implications on the time taken by the experiment and on its yield. Starting from ~ 6 pmol of each fragment, we typically obtain ~ 0.15 pmol of labeled hemicatenanes in a week's work, which is actually well sufficient for many further experiments. Therefore a study of the biochemical properties of DNA hemicatenanes is now possible. Scaling up the preparation might also permit the use hemicatenanes as a novel building block in DNA nanotechnology.

An important early step in the protocol is the preparation of catenated single strand DNA circles. The method used is inspired from previous work by Bucka and Stasiak [24], the main difference being that it does not require any homology between the two nucleotide sequences, as we used a synthetic oligonucleotide to keep the two strands in close vicinity during circularization by ligation. This absence of homology should also minimize the possibility that the strands wind around each other in the single strand catenane. The possibility of such winding of the strands still exists, however, due either to unavoidable complementarity between very short sequences, or simply to random motion of the single strands in the course of ligation. This heterogeneity in the linking of the two single strand circles is currently being studied as a function of various parameters, especially the temperature of ligation and the structure of the linker oligonucleotide.

The unwinding of the two double strand circles in the hemicatenane is easy to study, and the results of Fig. 9 showed each circle to be underwound by an average of half a double helical turn in the hemicatenane. The first interpretation that comes to mind is that the insertion of a strand between the two strands of the other duplex opens the double helix by half a turn, which is in good agreement with an intuitive view of a hemicatenane. This will have to be further studied, however, as we have no information about the structural heterogeneity of the material under study: first, we may be dealing with a mixture of left-handed and right-handed hemicatenanes, and DNA unwinding may not be the same in both chiral forms; second, the small amount (~ 10%) of topoisomers-2 in circles released from hemicatenanes (best visible in Fig. 9, lane 9) may reflect the presence of a minor proportion of structures with reciprocal winding of the internal strands by more than one turn. In any case it seems very likely that a large majority of the material prepared consists in simple hemicatenanes.

The fact that the band of hemicatenanes runs as a double band on polyacrylamide gels can actually be explained when it is noticed that the topoisomers of the larger circle migrate as two distinct bands on gels, even when no chloroquine is added (see e.g. the double band of covalently closed circles in Fig. 8 lane 11): topoisomers 0 and -1 of the larger circle do not migrate with the same mobility, which is not the case with the smaller circle. Since the larger circle is released from hemicatenanes as a roughly equal mixture of both topoisomers, it seems likely that the migration of hemicatenanes as a double band is due to the different migration of these two topoisomers. This interpretation is confirmed by the fact that the double band of hemicatenanes becomes a single band upon nicking of the outside strand of the larger circle (Fig. 8, nicking by enzyme b, lane 3).

Hemicatenanes of covalently closed circles are perfectly stable, as expected. In contrast, nicking of either internal strand results in a quick dissociation of the structure, an instability which is probably energetically driven by the unwinding of the double helices at the junction that was discussed above. This instability, observed here after incubation for 1 hr at 37°C with the nicking endonuclease, is currently under study as a function of temperature, ionic conditions, and DNA intercalating agents.

The structure prepared here can now be used to test some of the many different aspects of hemicatenanes that were summarized in the Introduction. The mobility of the junction seems particularly interesting to study. It is already clear that the hemicatenane can move easily along the sequence, since dissociation upon nicking requires that the nick be precisely located at the junction, whereas if the site for the nicking enzyme was at the junction in the covalently closed structure it would certainly not be cut by the nicking enzyme. It will be very interesting to study whether the hemicatenane has preferential locations as a function of the nucleotide sequences at the junction. More generally, the method described here should allow one to begin testing the theoretical models that proposed a role for DNA hemicatenanes in the organization of the genome in loops and chromosomal domains, and a modulation of genetic expression as a function of the location of the loops along the genome sequence [22,23].
